# Impact of Care Interventions on the Survival of Patients with Cardiac Chest Pain

**DOI:** 10.3390/healthcare11121734

**Published:** 2023-06-13

**Authors:** Silmara Meneguin, Camila Fernandes Pollo, Murillo Fernando Jolo, Maria Marcia Pereira Sartori, José Fausto de Morais, Cesar de Oliveira

**Affiliations:** 1 Department of Nursing, Botucatu Medical School, Paulista State University—Unesp, São Paulo 18618687, SP, Brazil; camilapollo@hotmail.com (C.F.P.); murilloofj@gmail.com (M.F.J.); 2 Department of Plant Production, School of Agriculture, Paulista State University—Unesp, Botucatu 18610034, SP, Brazil; maria.mp.sartori@unesp.br; 3 Faculty of Mathematics, Federal University of Uberlândia, Uberlândia 38400902, MG, Brazil; jfmorais.ufu@hotmail.com; 4 Department of Epidemiology & Public Health, University College London, London WC1E 6BT, UK; c.oliveira@ucl.ac.uk

**Keywords:** chest pain, assistance, critical care, emergency medical services, nursing

## Abstract

Background: Chest pain is considered the second most frequent complaint among patients seeking emergency services. However, there is limited information in the literature about how the care provided to patients with chest pain, when being attended to in the emergency room, influences their clinical outcomes. Aims: To assess the relationship between care interventions performed on patients with cardiac chest pain and their immediate and late clinical outcomes and to identify which care interventions were essential to survival. Methods: In this retrospective study. We analyzed 153 medical records of patients presenting with chest pain at an emergency service center, São Paulo, Brazil. Participants were divided into two groups: (G1) remained hospitalized for a maximum of 24 h and (G2) remained hospitalized for between 25 h and 30 days. Results: Most of the participants were male 99 (64.7%), with a mean age of 63.2 years. The interventions central venous catheter, non-invasive blood pressure monitoring, pulse oximetry, and monitoring peripheral perfusion were commonly associated with survival at 24 h and 30 days. Advanced cardiovascular life support and basic support life (*p* = 0.0145; OR = 8053; 95% CI = 1385–46,833), blood transfusion (*p* < 0.0077; OR = 34,367; 95% CI = 6489–182,106), central venous catheter (*p* < 0.0001; OR = 7.69: 95% CI 1853–31,905), and monitoring peripheral perfusion (*p* < 0.0001; OR = 6835; 95% CI 1349–34,634) were independently associated with survival at 30 days by Cox Regression. Conclusions: Even though there have been many technological advances over the past decades, this study demonstrated that immediate and long-term survival depended on interventions received in an emergency room for many patients.

## 1. Background

Chest pain is considered the second most frequent complaint among patients seeking emergency services. On average, six million patients visit emergency centers on an annual basis due to chest pain [1]. This symptom, which can be cardiac or non-cardiac in origin, benign or life-threatening, requires prompt diagnosis and management [2].

Among cardiac diseases, acute coronary syndrome (ACS), the most common clinical symptom of coronary artery disease, is characterized by a set of manifestations of acute myocardial ischemia and is responsible for 1/5 of the causes of chest pain [3,4].

Initially, clinical manifestations of ACS occur following a decrease in local circulation due to luminal narrowing. This commonly presents as a pressure chest pain that radiates to the left or right upper limb or mandible and may be associated with cold sweating, nausea, abdominal pain, or lipothymia [5].

The published literature shows that less than 15% of ACS cases are correctly diagnosed [6]. In Canada, it is estimated that 4.6% of patients with acute myocardial infarction and 6.4% of cases with unstable angina are misdiagnosed [7]. In Brazil, the proportion of ACS patients who are diagnosed ranges from 2% to 10% [4].

Globally, heart disease has remained the leading cause of death for the last 20 years, and it represents 16% of total deaths from all causes [8]. In Brazil, besides being the main cause of death since 1960, heart disease contributes to 31% of all deaths [9,10].

These data illustrate the need for public health strategies that seek to mitigate the impact of these conditions that are the main cause of morbidity and mortality in developed and developing countries [11,12].

In the emergency room, patient care for such patients should be focused on clinical history, a survey of symptoms, research on related risk factors, physical examination, and requests for additional tests such as electrocardiogram and troponin, which should be tested every 6–12 h at least [13,14].

During this phase, nursing care is based on comprehensive care. Additionally, the nursing care plan developed in the acute stage of the disease must include the patient’s basic human needs [15]. The aim of this care plan is to contribute to stabilizing the patient, reducing morbidity and mortality, and preventing further complications [11].

However, there is limited information in the literature about how the care provided to patients with chest pain, when being attended to in the emergency room, influences their clinical outcomes. Extensive research has been focused on the care provided for other conditions, such as post-cardiac arrest and HIV, among others [12,16].

Based on this gap in the literature, this investigation was carried out to answer the following questions: Is the care provided to patients with chest pain in the emergency room related to clinical outcomes? Do the interventions provided by this care have an impact on patient survival?

This study set out to verify whether the interventions performed on patients with cardiac chest pain had an impact on their immediate and late clinical outcomes as well as to identify the care interventions that could be described as survival factors.

## 2. Methods

This descriptive, exploratory, and retrospective study was conducted at an Emergency Center of a public institution in the State of São Paulo, from February to October 2019.

Patients of both sexes, aged 18 or older, admitted to the emergency room of the Emergency Center and hospitalized for a period of 30 days or less with chest pain due to a cardiac etiology documented in the medical record were considered eligible for the study. Patients who were hospitalized for more than 30 days were excluded.

Initially, a survey of all the daily consultations was carried out in the emergency room of the emergency center, from January to June 2018. Following this, medical records of 274 patients who presented with chest pain were retrieved; 118 were then excluded because the chest pain was attributed to a cardiac cause and 3 were excluded as they had been hospitalized for more than 30 days. Ultimately, 156 patients with cardiac chest pain were included in this analysis.

Two data collection instruments were used; the first consisted of sociodemographic and clinical data, namely date of hospital admission, data collection, date of birth, sex, origin before the admission to the emergency room, personal history, or comorbidities, diagnostic or diagnostic hypothesis, treatment (double anti-aggregation, invasive or thrombolytic interventionist), complications throughout hospitalization, and clinical outcomes (death and survival).

The second instrument documented the nursing care given towards cardiac chest pain, which was listed following the standardization proposed by the Nursing Interventions Classification (NIC). This included the following variables: heart rate (HR) and pace, neurological status, liquid balance, bladder catheterization, 12-lead electrocardiogram (ECG), peripheral venous puncture (PVP), central venous puncture (CVP), control laboratory tests, whether a chest x-ray was done, non-invasive blood pressure (NIBP), invasive blood pressure (IBP), pulse oximetry, arterial blood gases, partial oxygen pressure (PaO_2_), oxygen therapy, peripheral perfusion, antiarrhythmic therapy, cardiac auscultation, pulmonary auscultation, 2-hourly change of position, use of anticoagulants, medications dispensed to relieve/prevent pain and ischemia, thrombolytic therapy, basic and advanced life support measures (BLS and ACLS), institution of oral nutrition, institution of enteral nutrition, transfer to intensive care unit (ICU), transfer to service hemodynamics, and use of vasoactive drug and blood transfusion [17].

To answer the study’s objectives, patients were divided into two groups: Group 1 (G1) consisted of patients who were hospitalized for only 24 h, and Group 2 (G2) consisted of patients who were hospitalized for a period ranging from 25 h to 30 days. Groups 1 and 2 were not different. Their separation was done for survival measurement purposes.

Descriptive analyses were performed, initially. Pearson’s chi-square test or Fisher’s exact test were used to compare the interventions performed in both patient groups.

Kaplan-Meir survival curves were calculated for each type of care/intervention performed considering survival at 24 h and 30 days. The data were assessed after 30 days of follow-up with the possible outcome being discharge or death. The comparison of survival at 24 h was done using a comparison test like the Chi-square test. A Cox regression model was adjusted for survival time considering the different types of care provided using the Stepwise method to select the interventions which were more correlated to survival time.

PCA (Principal Component Analysis) was used to assess the correlation between the interventions performed and survival. The analysis used the R software version 3.6.2 and the Statistical Package for the Social Sciences (SPSS) version 20.0, Windows platform.

This research was approved by the Research Ethics Committee (protocol no. 4.011.394.).

## 3. Results

Based on the inclusion criteria, 153 participants were included in the study sample; of those participants, 23 were hospitalized for only 24 h (Group G1), and 130 were hospitalized for a period ranging from 25 h to 30 days (Group G2). Table 1 shows the participants’ sociodemographic and clinical characteristics for G1 and G2 groups and for all participants.

Most of the participants were male 99 (64.7%), with a mean age of 63.1 years, had comorbidities (88.9%), among which high blood pressure 107 (69.9%), dyslipidemia 66 (43.1%), and diabetes 57 (37.3%) stood out as the most prevalent. Most participants had a history of smoking 83 (54.2%) and on average, received six interventions. Around one-tenth (10%) of the participants died. With the exception of the variables days of hospitalization and outcome, no statistically significant difference was identified between the statistics when comparing groups G1 and G2. In the case of the variable days of hospitalization, the result was as expected and in the case of the outcome variable, the significance was borderline.

Table 2 describes the information related to chest pain, diagnosis, and treatment for G1 and G2 groups and for all participants. The most prevalent chest pain was precordial (89.5%) associated with sweating (12.4%) and that radiated (56.9). The duration of pain for most participants (37.3%) was less than one hour and the predominant medical diagnosis was acute myocardial infarction (68.6%). Most patients were managed conservatively with the use of mainly double antiplatelet therapy (68.0%) and invasive interventional therapy (26.8%). No statistically significant association was identified between the variables and groups G1 and G2.

Table 3 describes the nursing interventions provided to the study participants. The most prevalent interventions in both groups were performing a 12-lead ECG (96.7%), monitoring pace and HR (84.3%), control laboratory tests (82.4%), and peripheral venous puncture (70.6%). On the other hand, a central venous puncture was performed more frequently in G2 than in G1 (31.0% vs. 13.3%; *p* = 0.025, respectively). Furthermore, a transfer to ICU was also more common in G2 when the groups were compared (0.00% vs. 30%; *p* = 0.002).

Table 4 displays the types of care that were associated with survival rates using the Kaplan-Meir method at 24 h and 30 days. Survival at 24 h was significantly associated with monitoring neurological status (*p* < 0.0001), central venous catheter (*p* = 0.0006), non-invasive blood pressure monitoring (*p* = 0.0074), pulse oximetry (*p* = 0.0007), and monitoring peripheral perfusion (*p* ≤ 0.0001). Survival at 30 days was significantly associated with vesical catheterization (*p* = 0.0093), central venous catheter (*p* ≤ 0.0001), non-invasive blood pressure monitoring (*p* = 0.0019), pulse oximetry (*p* = 0.019), peripheral perfusion monitoring (*p* = 0.0006), oxygen therapy (*p* = 0.0139), BLS/ACLS (*p* < 0.0001), use of vasoactive drug (*p* = 0.0002), and blood transfusion (*p* < 0.0001)

Table 5 shows the results of the Cox regression analysis. There was a statistically significant relationship between patient survival and the following interventions: central venous puncture (*p* ≤ 0.0001; HR = 7.69: 95% CI 1.853–31.905), peripheral perfusion monitoring (*p* ≤ 0.0001; HR = 6.835; 95% CI 1.349–34.634), BLS and ACLS (*p* = 0.0145; HR = 8.053; 95% CI = 1.385–46.833), and blood transfusion (*p* = 0.0077; HR = 34.367; 95% CI = 6.489–182.106). PCA (Principal Component Analysis).

**Figure 1:**
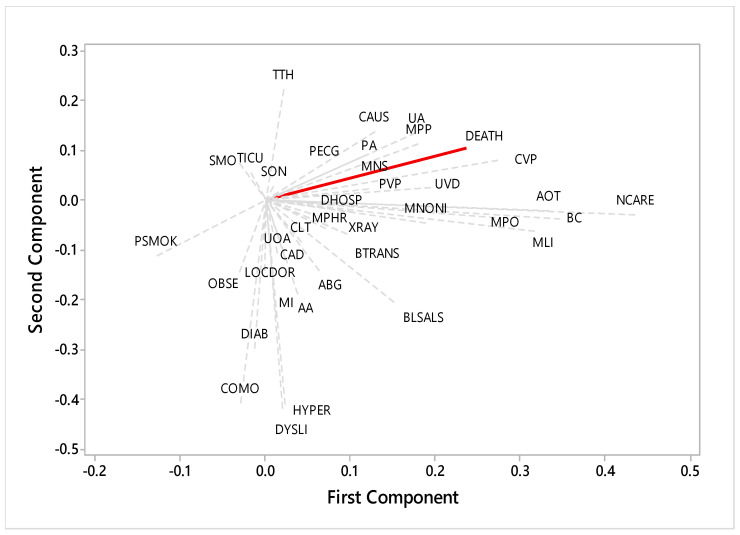
Principal Component Analysis (Legend: Comorbidities: COMO; Obesity: OBSE; Smoking: SMO; Previous smoking: PSMOK; Dyslipidemia: DYSLI; Diabetes: DIAB; Hypertension: HYPER; Myocardial infarction: MI; Coronary artery disease: CAD; Monitor pace and Heart Rate: MPHR; Monitor neurological state: MNS/MLI; Bladder catheterization: BC; Performing a 12-lead ECG: PECG; Peripheral venous puncture: PVP; Central venous puncture: CVP; Control laboratory tests: CLT; Getting X-ray: XRAY; Monitor non-invasive blood pressure: MNONI; Monitor pulse oximetry: MPO; Arterial blood gases: ABG; Administer oxygen therapy: AOT; Monitor peripheral perfusion: MPP; Use of antiarrhythmic: UOA; Cardiac auscultation: CAUS; Pulmonary auscultation: PA; Administer anticoagulants: AA; BLS and ALS:BLSALS; Start oral nutrition: SON; Transfer to ICU: TICU; Transfer to hemodynamics: TTH; Use of vasoactive drug: UVD; Blood transfusion: BTRANS; Number care: NCARE; Death: DEATH; Days of hospitalization: DHOSP).

In Figure 1 we can observe from the PCA (Principal Component Analysis) that the variables comorbidities, obesity, previous smoking, and transfer to ICU showed a negative correlation with death (i.e., no detectable/direct effect). However, the remaining variables showed a positive effect on death (i.e., red line). The survival presents a greater correlation with the interventions that are in the second quadrant of the figure (right).

## 4. Discussion

The nurse’s role in the emergency room is crucial in determining the differential diagnosis of chest pain and the prognosis of a patient presenting with chest pain of cardiac origin which requires prompt and specific care. In this study, there was a predominance of older male participants who were hospitalized for an average of six days. This demographic is similar to that in a study conducted in another state of Brazil for patients with chest pain complaints who were admitted to the emergency room [18]. Additionally, in the literature, among patients with acute chest pain seen in the emergency room, women are less likely to have AMI or be diagnosed with ischemic heart disease [19].

With regards to comorbidities, arterial hypertension, dyslipidemia, and diabetes stood out as the most prevalent comorbidities, since they are considered classic risk factors for the onset of cardiovascular diseases. A recent study carried out among 80 patients admitted after AMI at the University Hospital of Sarajevo showed that the incidence of diabetes mellitus and obesity was significantly higher in patients aged over 45 years. Conversely, other risk factors, such as hypertension and cholesterol, were prevalent among all age groups [20].

In this study, precordial radiating pain of less than one hour’s duration was the most common symptom at the time of admission. Cardiac chest pain usually lasts for a few minutes, whereas angina lasts for 2 to 10 min and AMI for more than 20 min. A sudden or continuous pain lasting several hours is rarely angina [21].

The most common diagnoses were myocardial infarction followed by unstable angina. This concurs with data from the national epidemiological profile that shows that 15% to 30% of patients with chest pain are diagnosed with acute myocardial infarction with ST-segment elevation or unstable angina [11].

Close to 10% of participants seen in the emergency room died. Studies have shown that 40–65% of deaths from AMI take place within the first hour of presentation while 80% occur within the first 24 h [22]. A high mortality rate within this timeframe could be attributed to arrhythmias such as ventricular fibrillation leading to cardiorespiratory arrest.

The ACC/AHA Joint Committee on Clinical Practice Guidelines recently developed and published a guideline for evaluation and diagnosis of chest pain. The intent of the new guideline is to outline a framework for evaluation of acute or stable chest pain syndromes or other anginal equivalents in various clinical settings, but especially in emergency departments, with emphasis on identification of ischemic and other potentially high-risk etiologies [23].

In the TIMI risk score for AMI with ST-segment elevation validation study in Porto Alegre Brazil, the mortality rate of 602 patients with a maximum age limit of 65 years was 8.6%. In the international literature, mortality rates after AMI range from 7.6 to 24% [24,25,26].

However, there are gender differences in the prognosis of AMI. A survey of 2042 patients revealed that in the short term (28 days), the prognosis of AMI is similar for both sexes; however, in the long term (7 years), the prognosis of male patients with AMI is worse when compared to women [27].

The most common nursing care provided was performing 12-lead ECG; however, this did not differ by patient group. It is well-established in the literature that the “door-ECG” time interval should not exceed ten minutes. Moreover, all patients with suspected ACS should have their heart pace and heart rate monitored at the initial assessment to detect cardiac arrhythmias and conduction disorders, which are frequent during the first hours of AMI. In clinical practice, it is possible that there may have been recording mistakes during this investigation due to the dynamics of care in this type of service. However, we did not evaluate for this [11].

Another frequent intervention performed by the nursing care team was to check laboratory tests. From the blood collection, it is possible to identify the biochemical markers of myocardial damage, such as troponin, CKMB, and myoglobin, which are essential for confirming a diagnosis of infarction. There is a direct association between the elevation of such biochemical markers and the risk of cardiac events; troponin is considered the most specific marker for myocardial ischemia [28].

The peripheral venous puncture for rapid drug administration, for laboratory tests, and prior to intravenous therapy was also a routine procedure in clinical practice.

After comparing the interventions performed on the two patient groups, patients in G2 were more likely to have had a central venous puncture and to be transferred to the ICU. It is possible that this care was only provided to patients who remained hospitalized, probably due to clinical complications, hemodynamic instability, and the need for vasoactive drugs.

The present study showed, after analyzing the type of patient care provided based on the length of time investigated (i.e., immediate (24 h) and delayed (30 days)), that central venous catheter, non-invasive blood pressure monitoring, pulse oximetry, and monitoring peripheral perfusion were associated with survival in both groups of patients. Patients in pain or showing signs and symptoms of respiratory failure should, therefore, receive oxygen if their oxygen saturation is below 94%. This also justifies their rigorous monitoring [11].

With regards to the wide use of pulse oximetry, it is important for nurses to have good knowledge of interpretation of its parameters to avoid any errors and, ultimately, prevent any risk to the patient [29]. In addition, nurses should understand that although pulse oximetry has a key role in the oxygen level assessment, it is not the most adequate procedure to assess a patient’s ventilation capacity [30]. The pulse oximetry only measures the percentage of saturated hemoglobin with oxygen. Both the hemoglobin level and SpO_2_ are needed to accurately interpret the oxygen level available for perfusion [31].

Based on the Cox regression analysis performed to answer the second specific objective of this study, monitoring peripheral perfusion and conducting peripheral venous puncture, BLS, ACLS, and blood transfusion positively contributed to increasing a patient’s chance of survival at 30 days.

The use of the central venous catheter was a procedure that influenced survival positively in all analyses, corroborating the findings from clinical practice since this type of catheter is also used in the infusion of vasoactive drugs.

Our results regarding both BLS and ACLS maneuvers are in accordance with previous studies showing that adopting an immediate intervention such as cardiopulmonary resuscitation of individuals with cardiac arrest is crucial to increase their survival rate [32].

A previous study conducted with 35.065 patients who suffered cardiac arrest outside a hospital environment showed that advanced life support care was associated with survival until hospital discharge when provided initially or within six minutes from the BLS, Advanced life support care, with or without basic life support, was associated with an increased return of spontaneous circulation [33].

### Limitations

There were some limitations. Incomplete documentation of patient care provided in the emergency room impacted the comprehensiveness of our analysis. Furthermore, the patient’s severity score, a vital piece of information due to mortality risk prediction, was not evaluated.

Furthermore, limited information in the literature regarding the types of interventions given to patients with chest pain made it difficult to compare our results to that of published literature. Nevertheless, more research should be conducted in this area in which nursing plays a major role.

## 5. Conclusions

Even though there have been many technological advances over the past decades, this study demonstrated that immediate and long-term survival depended on interventions received in an emergency room for many patients.

Central venous catheter, non-invasive blood pressure monitoring, pulse oximetry and monitoring peripheral care interventions were associated to survival at 24 h and 30 days. Advanced and basic support life support, blood transfusion, central venous catheter, and monitoring peripheral perfusion influenced independently the survival rate of participants.

## Figures and Tables

**Table 1:** Sociodemographic, clinical, and care-related characteristics of study participants. Botucatu, SP, Brazil, 2020.

Variable	G1 (n = 23)	G2 (n = 130)	Total (n = 153)	*p*
Sex				
Male	16 (69.6)	83 (63.8)	99 (64.7)	
Female	7 (30.4)	47 (36.2)	54 (35.3)	0.597 ^1^
Age (years)				
Mean (±SD)	60.2 ± 14.2	63.6 ± 13.0	63.1 ± 13.2	0.289 ^2^
Days of hospitalization				
Mean (±SD)	2.9 ± 3.6	7.7 ± 6.1	7.0 ± 6.0	<0.001
Comorbidities				
Yes	21 (91.3)	115 (88.5)	136 (88.9)	0.512 ^4^
Arterial hypertension				
Yes	17 (73.9)	90 (69.2)	107 (69.9)	0.652 ^1^
Dyslipidemia				
Yes	13 (56.5)	74 (56.9)	87 (56.9)	0.971 ^1^
Diabetes Mellitus				
Yes	16 (69.6)	80 (63.8)	96 (62.7)	0.597 ^1^
History of infarction				
Yes	3 (13.0)	21 (16.2)	24 (15.7)	0.494 ^4^
Coronary artery disease				
Yes	3 (13.0)	16 (12.3)	19 (12.4)	0.572 ^4^
Obesity				
Yes	2 (8.7)	10 (7.7)	12 (7.8)	0.566 ^4^
History of smoking				
Yes	10 (43.5)	73 (56.2)	83 (54.2)	0.261 ^1^
Smoking				
Yes	5 (21.7)	30 (23.1)	35 (22.9)	0.888 ^1^
Number of interventions				
Mean (±SD)	6.3 ± 2.7	6.0 ± 2.1	6.0 ± 2.2	0.903 ^3^
Outcome				
Survivals	18 (78.3)	120 (92.3)	138 (90.2)	
Deaths	5 (21.7)	10 (7.7)	15 (9.8)	0.052 ^4^

^1^ Pearson’s chi-square test. ^2^ *t*-test for independent samples. ^3^ Mann-Whitney U test. ^4^ Fisher’s exact test.

**Table 2:** Characterization of chest pain, associated symptoms, medical diagnosis, and treatment. Botucatu, 2020.

Variable	G1 (n = 23)	G2 (n = 130)	Total (n = 153)	*p* *
Pain site				
Precordial	20 (87.0)	119 (91.5)	137 (89.5)	
Epigastric	3 (13.0)	5 (3.8)	8 (5.2)	
Precordial and Epigastric	0 (0.0)	6 (4.6)	6 (3.9)	0.118
Pain irradiation				
Yes	15 (65.2)	72 (55.4)	87 (56.9)	0.380
Duration of pain (in hours)				
<1	14 (60.9)	43 (33.1)	57 (37.3)	
1 to 3	3 (13.0)	31 (23.8)	34 (22.2)	
4 to 23	3 (13.0)	34 (26.2)	37 (24.2)	
>24	3 (13.0)	22 (16.9)	25 (16.3)	0.084
Presence of associated symptoms				
Yes	12 (52.2)	67 (51.5)	79 (51.6)	0.955
Associated symptoms				
Sweating	4 (17.4)	15 (11.5)	19 (12.4)	
Nausea and Vomiting	1 (4.3)	10 (7.7)	11 (7.2)	
Dyspnea	0 (0.0)	10 (7.7)	10 (6.5)	
Fatigue	0 (0.0)	4 (3.1)	4 (2.6)	
Other	11 (47.8)	63 (48.5)	74 (48.4)	0.411
Medical diagnostics				
Infarction with ST-segment elevation	11 (47.8)	63 (48.50	74 (48.3)	
Infarction without ST-segment elevation	5 (21.7)	36 (27.7)	41 (26.8)	
Unstable angina	6 (26.1)	23 (17.7)	29 (18.9)	
Stable angina	1 (4.3)	2 (1.5)	3 (2.0)	
Cardiac failure	0 (0.0)	3 (2.3)	3 (2.0)	
Angina secondary to tachyarrhythmia	0 (0.0)	3 (2.3)	3(2.0)	0.725
Treatment				
Double anti-aggregation	19 (82.6)	85 (65.4)	104 (68.0)	
Interventional	3 (13.0)	38 (29.2)	41 (26.8)	
Thrombolytic	1 (4.3)	7 (5.4)	8 (5.2)	0.246

* Pearson Chi-square test.

**Table 3:** Interventions performed on study participants in the emergency room concerning groups of participants. Botucatu, SP, Brazil.

Interventions	G1 (n = 23)	G2 (n = 130)	Total (n = 153)	*p*
1. Monitor pace and heart rate	21 (91.3)	108 (83.1)	129 (84.3)	0.533 ^1^
2. Monitor neurological state	0 (0.0)	6 (4.6)	6 (3.9)	0.592 ^1^
3. Monitor liquid intake	3 (13.0)	10 (7.7)	13 (8.5)	0.416 ^1^
4. Bladder catheterization	3 (13.0)	10 (7.7)	13 (8.5)	0.416 ^1^
5. Performing a 12-lead ECG	22 (95.7)	126 (96.9)	148 (96.7)	0.562 ^1^
6. Peripheral venous puncture	13 (56.5)	95 (73.1)	108 (70.6)	0.108 ^2^
7. Central venous puncture	2 (1.5)	3 (13.0)	5 (3.3)	0.025 ^1^
8. Control laboratory tests	19 (82.6)	107 (82.3)	126 (82.4)	1.000 ^1^
9. Get X-ray	12 (52.2)	50 (38.5)	62 (40.5)	0.217 ^2^
10. Monitor non-invasive blood pressure	7 (30.4)	26 (20.0)	33 (21.6)	0.277 ^1^
11. Monitor pulse oximetry	3 (13.0)	10 (7.7)	13 (8.5)	0.416 ^1^
12. Arterial blood gases	0 (0.0)	3 (2.3)	3 (2.0)	1.000 ^1^
13. Administer oxygen therapy	4 (17.4)	14 (10.8)	18 (11.8)	0.479 ^1^
14. Monitor peripheral perfusion	2 (8.7)	2 (1.5)	4 (2.6)	0.108 ^1^
15. Use of antiarrhythmic	1 (4.3)	12 (9.2)	13 (8.5)	0.693 ^1^
16. Cardiac auscultation	3 (13.0)	12 (9.2)	15 (9.8)	0.702 ^1^
17. Pulmonary auscultation	3 (13.0)	19 (14.6)	22 (14.4)	1.000 ^1^
18. Administer anticoagulants	11 (47.8)	50 (38.5)	61 (39.9)	0.398 ^2^
19. Use of analgesics medication	1 (4.3)	11 (8.5)	12 (7.8)	0.695 ^1^
20. BLS and ALS *	1 (4.3)	2 (1.5)	3 (2.0)	0.389 ^1^
21. Start oral nutrition	0.00	5 (3.8)	5 (3.3)	1.000 ^1^
22. Transfer to ICU	0 (0.00)	39 (30.0)	39 (25.5)	0.002 ^2^
23. Transfer to hemodynamics	8 (34.8)	38 (29.2)	46 (30.1)	0.592 ^2^
24. Use of vasoactive drug	5 (21.7)	25 (19.2)	30 (19.6)	0.779 ^1^
25. Blood transfusion	1 (4.3)	1 (0.8)	2 (1.3)	0.279 ^1^

^1^ Fisher’s exact test. ^2^ Pearson’s chi-square test. * Basic Life Support and Advanced Life Support.

**Table 4:** Survival at 24 h and 30 days and the different types of care/interventions investigated. Botucatu, SP, Brazil.

Type of Care/Intervention	Survival Time			
	24 h	30 Days	*p* *	*p* **
1. Monitor pace and heart rate	1	0.2018	0.7222	0.6533
2. Monitor neurological state	0.966	0.1432	<0.0001	0.7051
3. Monitor liquid intake	0.9786	1.0146	0.0795	0.3138
4. Bladder catheterization	0.9786	6.7704	0.0795	0.0093
5. Performing a 12-lead ECG	1	0.3999	1	0.5271
6. Peripheral venous puncture	0.9556	0.4392	0.9766	0.5075
7. Central venous puncture	0.9797	20.4478	0.0006	<0.0001
8. Control laboratory tests	0.9259	0.7428	0.2953	0.3888
9. Get X-ray	0.967	0.9471	1	0.3305
10. Monitor non-invasive blood pressure	0.9917	9.6303	0.0074	0.0019
11. Monitor pulse oximetry	0.9857	5.5026	0.0007	0.019
12. Arterial blood gases	0.9667	0.2087	1	0.6478
13. Administer oxygen therapy	1	6.0546	1	0.0139
14. Monitor peripheral perfusion	0.9799	11.8516	<0.0001	0.0006
15. Use of antiarrhythmic	0.9797	1.2963	1	0.2549
16. Cardiac auscultation	0.971	0.6301	0.988	0.4273
17. Pulmonary auscultation	0.9695	0.0624	1	0.8027
18. Administer anticoagulants	0.9565	1.2973	0.6468	0.2547
19. Use of analgesics	0.9645	1.3717	1	0.2415
20. BLS and ALS ***	0.9733	28.7675	0.1876	<0.0001
21. Start oral nutrition	0.9662	0.5221	1	0.47
22. Transfer to ICU	0.9561	1.2891	0.419	0.2562
23. Transfer to hemodynamics	0.972	0.1492	1	0.6993
24. Use of vasoactive drug	0.9837	13.6671	0.0818	0.0002
25. Blood transfusion	0.9735	28.2419	0.0819	<0.0001

* *p*-value for the comparison of the survival rate at 24 h. ** 30-day survival assessment using the log-rank test. *** Basic Life Support and Advanced Life Support.

**Table 5:** Cox regression of the types of intervention associated to a higher survival rate in 30 days. Botucatu, SP, Brazil.

Interventions	*p*	OR	95% CI	
			Minimum	Maximum
Central venous puncture	<0.0001	7.69	1.853	31.905
Monitor peripheral perfusion	<0.0001	6.835	1.349	34.634
BLS and ACLS	0.0145	8.053	1.385	46.833
Blood transfusion	0.0077	34.376	6.489	182.106

## Data Availability

The data that support the findings of this study are available on request from the corresponding author. The data are not publicly available due to restrictions (e.g., their containing information that compromise the privacy of research participants). All listed authors meet the authorship criteria, and all authors agree with the content of the manuscript.
